# Flu Days

**DOI:** 10.3201/eid2212.AD2212

**Published:** 2016-12

**Authors:** Peter Makuck

**Keywords:** influenza, flu, Flu Days, poem

Shivering, you drag yourself,
as if gun-shot, to the living room,
to the old movie channel, to a Bogart festival,
your mind fogged over (like the street on the screen)
edging toward feverish sleep when Bogey snarls
at Ida Lupino: “Of all the 14-carat saps…”
Hours later when you wake, he’s smacking Peter Lorre:
“When you’re slapped, you’ll take it and like it!”
And as if cuffed, you black out, head pounding, and come to
upon Ingrid Bergman and “You must remember this,”
before fading again, then back to Bogey hacked to death
by Bedoya’s machete,
all that gold dust blown away
with the whole bloody day, everything gone—gone black
as your living room windows— those previews of The Big Sleep.

